# Risk factors and outcomes of lower respiratory tract infections after traumatic brain injury: a retrospective observational study

**DOI:** 10.3389/fmed.2023.1077371

**Published:** 2023-04-17

**Authors:** Eder Caceres, Juan C. Olivella, Miguel Yanez, Emilio Viñan, Laura Estupiñan, Natalia Boada, Ignacio Martin-Loeches, Luis Felipe Reyes

**Affiliations:** ^1^Unisabana Center for Translational Science, Universidad de La Sabana, Chía, Colombia; ^2^Neurocritical Care Division, Critical Care Department, Clínica Universidad de La Sabana, Chía, Colombia; ^3^Centro de Investigación Biomédica en Red de Enfermedades Respiratorias (CIBERES), Instituto de Salud Carlos III, Madrid, Spain; ^4^Multidisciplinary Intensive Care Research Organization (MICRO), Department of Intensive Care Medicine, St. James's University Hospital, Dublin, Ireland; ^5^Critical Care Department, Trinity Centre for Health Sciences, Dublin, Ireland; ^6^Hospital Clínic, IDIBAPS, Universidad de Barcelona, Barcelona, Spain; ^7^Facultad de Medicina, Universidad de La Sabana, Chía, Colombia; ^8^Pandemic Science Institute, University of Oxford, Oxford, United Kingdom

**Keywords:** traumatic brain injury, hospital-acquired pneumonia, ventilator-associated tracheitis, ventilator-associated pneumonia, multiple trauma, acute brain injury

## Abstract

**Background:**

Traumatic brain injury (TBI) is a public health problem with a high burden in terms of disability and death. Infections are a common complication, with respiratory infections being the most frequent. Most available studies have addressed the impact of ventilator-associated pneumonia (VAP) after TBI; therefore, we aim to characterize the hospital impact of a broader entity, lower respiratory tract infections (LRTIs).

**Methods:**

This observational, retrospective, single-center cohort study describes the clinical features and risk factors associated with LRTIs in patients with TBI admitted to an intensive care unit (ICU). We used bivariate and multivariate logistic regressions to identify the risk factors associated with developing LRTI and determine its impact on hospital mortality.

**Results:**

We included 291 patients, of whom 77% (225/291) were men. The median (IQR) age was 38 years (28–52 years). The most common cause of injury was road traffic accidents 72% (210/291), followed by falls 18% (52/291) and assault at 3% (9/291). The median (IQR) Glasgow Coma Scale (GCS) score on admission was 9 (6–14), and 47% (136/291) were classified as severe TBI, 13% (37/291) as moderate TBI, and 40% (114/291) as mild TBI. The median (IQR) injury severity score (ISS) was 24 (16–30). Nearly 48% (141/291) of patients presented at least one infection during hospitalization, and from those, 77% (109/141) were classified as LRTIs, which included tracheitis 55% (61/109), ventilator-associated pneumonia (VAP) 34% (37/109), and hospital-acquired pneumoniae (HAP) 19% (21/109). After multivariable analysis, the following variables were significantly associated with LRTIs: age (OR 1.1, 95% CI 1.01–1.2), severe TBI (OR 2.7, 95% CI 1.1–6.9), AIS thorax (OR 1.4, 95 CI 1.1–1.8), and mechanical ventilation on admission (OR 3.7, 95% CI 1.1–13.5). At the same time, hospital mortality did not differ between groups (LRTI 18.6% vs. No LRTI 20.1%, *p* = 0.7), and ICU and hospital length of stay (LOS) were longer in the LRTI group (median [IQR] 12 [9–17] vs. 5 [3–9], *p* < 0.01) and (median [IQR] 21 [13–33] vs. 10 [5–18], *p* = 0.01), respectively. Time on the ventilator was longer for those with LRTIs.

**Conclusion:**

The most common site/location of infection in patients with TBI admitted to ICU is respiratory. Age, severe TBI, thoracic trauma, and mechanical ventilation were identified as potential risk factors. LRTI was associated with prolonged ICU, hospital stay, and more days on a ventilator, but not with mortality.

## Background

Traumatic brain injury (TBI) is a public health issue and a leading cause of mortality and disability in the younger population. Additionally, TBI impacts the quality of life of older adults, who usually have a reduced capacity to recover after these events (1, 2). TBI is associated with changes in the immune system mediated through inflammatory and autonomic pathways (3, 4) that seem to increase the susceptibility to infections during and after hospitalization (3–5). Among individuals with TBI who suffer nosocomial infections, a frequent source is the respiratory system or what we will denominate lower respiratory tract infections (LRTIs), including ventilator-associated pneumoniae (VAP), ventilator-associated tracheitis (VAT), and healthcare-associated pneumonia (HAP) (3, 6).

The most frequent sources of infection in previous cohorts of TBI have been respiratory and urinary, followed by surgical site infections. Regarding respiratory infections, a greater proportion of studies have focused on VAP. The frequency of VAP in these studies ranges from 31 to 47%, and some of them have found a positive correlation between VAP and several outcomes, including longer hospital stays and higher rates of mortality and disability. The severity of TBI, chest trauma, smoking history, drug abuse, and interventions, such as transfusions, sedation, and the need for a tracheostomy, are associated with VAP (7–11).

Other types of LRTIs might play a relevant role in the hospital course and outcomes of these patients (10). LRTIs could be considered a continuum spectrum of a single disease and, therefore, it might be valuable to describe its epidemiology and associated factors (12–14). This would allow a better understanding of this phenomenon and, accordingly, the development of measures to prevent and manage these complications. By identifying potential risk factors, we can highlight areas of care susceptible to improvement. In this study, our objectives are (a) to describe the epidemiology of lower respiratory tract infections (LRTIs) in our TBI cohort, (b) to determine factors associated with LRTI, and (c) to determine whether LRTI is associated with clinical outcomes (mortality, LOS).

## Materials and methods

This is a retrospective, observational, single-center cohort of patients with traumatic brain injury (TBI) admitted to the intensive care unit (ICU). Using the electronic medical record, we searched for patients admitted to the ICU from August 2009 to December 2019 with the diagnosis of TBI. Once selected, the diagnosis was confirmed, and data were validated through a medical chart review. The Ethics Committee of Clínica Universidad de La Sabana approved this study and waived the need for informed consent as only routinely collected clinical data were recorded.

### Data collection and storing

One or more dedicated and trained physicians collected clinical data through a medical chart review. These data included demographics, medical history, injury severity, and characteristics during the hospital stay. Data were collected using a Case report form (CRF) built on Research Electronic Data Capture (REDCap, version 8.11.11, Vanderbilt University, Nashville, Tenn.) hosted by the Universidad de Sabana. All study data were de-identified and stored securely by the Translational Science in Infectious Diseases and Critical Care Research Group at Universidad de La Sabana.

### Definitions

Lower respiratory tract infection (LRTI) is used as a broad term that includes hospital-acquired pneumoniae (HAP), ventilator-associated pneumoniae (VAP), and ventilator-associated tracheobronchitis (VAT). These entities were diagnosed using the definition of IDSA/ATS guidelines (15). The IDSA guideline criteria were also followed to assess the diagnosis of other infectious complications, including catheter-associated urinary tract infection (CA-UTI), surgical site infection (SSI), and catheter-related bloodstream infection (CRBSI) (16–18). TBI severity was determined using the Glasgow Coma Scale (GCS). Subjects with mild head injury (GCS 13–15) were included in this cohort when they were admitted to ICU; usually, this occurs when there is a risk of clinical deterioration, other body part injuries, and/or comorbid conditions. The severity of trauma and body regions compromised was established using the Abbreviated Injury Score (AIS) and the Injury Severity Score (19). ISS is the sum of the squares of the highest AIS scores in the three most severely injured regions; it ranges from 1 to 75; the higher the score, the more severe the injury. Isolated TBI was defined as AIS head ≥3 and injury to any other region with AIS < 3. Patients with TBI and multiple traumas had AIS head ≥3 and trauma to any other body region with AIS ≥ 3.

### Outcomes

The primary outcome was the diagnosis of LRTI during hospitalization. Clinical data were analyzed, and predictors of this primary outcome were identified. The secondary outcomes were hospital survival and the length of ICU and hospital stay.

### Statistical analysis

We excluded patients for whom information on discharge or clinical severity was missing. The Shapiro–Wilk test was used to detect departures from normality, and numerical variables were reported as mean (DS) or median (IQR) according to distribution. Categorical data are reported as numbers and percentages. Logistic regression models were used to explore the predictor variables for the primary outcome. Potential predictors for the primary outcome were identified in a univariate analysis. Then, a multivariable model was constructed adjusting for TBI severity, the severity of the trauma, and illness severity scores (APACHE II, SOFA), with results reported as odds ratios (95% CI). For testing the goodness of fit, we used the Hosmer–Lemeshow test. In secondary outcomes analyses, differences in mortality and length of stay between subjects with and without LRTIs were analyzed using the chi-square test for differences in proportions and the Mann–Whitney *U*-test for non-parametric data. A two-tailed *p*-value of 0.05 or less was used to define statistical significance. Statistical analyses were performed using R (version 4.2.1) and Studio (version 2022.07.0) as the integrated development environment.

## Results

From August 2009 to December 2019, 291 patients were enrolled. The baseline and clinical characteristics of the patients are presented in Table 1. The median (IQR) age was 38 (28–52) years, and men accounted for 77% (225/291) of the TBI admissions to the ICU. Road traffic accidents were the leading cause of TBI at 72% (210/291), followed by falls 18% (52/291) and assault at 3% (9/291). Median (IQR) GCS on admission was 9 (6–14), and 47% (136/291) were classified as severe TBI, 13% (37/291) as moderate TBI, and 40% (114/291) as mild TBI. Isolated TBI accounted for 21% (63/291), while the rest of the patients had associated injuries to at least one body region with AIS ≥ 3. In terms of lesions in other parts of the body, the more frequently compromised (AIS ≥ 3) were the thorax 29% (86/291), limbs 28% (84/291), and abdomen 14% (49/291). The median stay in the ICU was 7 (IQR, 4–13), and the median (IQR) hospital stay was 13 days (7–25 days). The frequency of patients requiring invasive ventilatory support was 83% (244/291), and the median (IQR) time on a ventilator was 5 days (3–9 days). Hospital mortality for this cohort was 19% (56/291), and survival time to death was 7 days (IQR, 4–13).

**Table 1:** Baseline and clinical characteristics of patients.

**Demographic characteristics**	***n =*** **291**
Age, median (IQR)	38 (28-52)
Male sex *n* (%)	225 (77)
**Cause of injury**	***n*** **(%)**
Road accident	210 (72)
Fall	52 (18)
Assault	9 (3)
Blast	3 (1)
Other	16 (5)
**Clinical presentation**	***n*** **(%)**
**GCS score**	
Mild (3–15)	114 (40)
Moderate (9–12)	37 (13)
Severe (3–8)	136 (47)
**AIS head score**	
Mild injury (1–2)	84 (29)
Moderate (3)	75 (25)
Severe (4–6)	132 (45)
**Injury type**	
Blunt	272 (93)
Penetrating	19 (7)
Injury severity score median (IQR)	24 (16–30)
**Severity of trauma**	***n*** **(%)**
ISS 1–15	63 (21)
ISS 16–24	93 (32)
ISS ≥ 25	125 (47)
AIS thorax ≥ 3	86 (29)
AIS limbs ≥ 3	84 (28)
AIS abdomen ≥ 3	49 (14)
AIS spine ≥ 3	30 (8)
AIS neck ≥ 3	15 (5)
APACHE II median (IQR)	13 (8–17)
SOFA	5 (3–7)

The major extracranial injury was defined as any non-head AIS score of ≥3. Mild trauma was defined as ISS of 1–15, moderate trauma ISS of 16-−24, and severe trauma ISS of ≥25. ISS, Injury Severity Score; AIS, Abbreviated Injury Score; GCS, Glasgow Coma Scale.

Infection complications were present in 48% (141/291) of patients, and from those, 77% (109/141) corresponded to LRTIs, 14% (20/141) to CA-UTI, 15% (22/141) to SSI, and 9% (14/141) to CRBSI. Regarding absolute frequency, 38% (109/291) had an LRTI, 7% (20/291) had a CA-UTI, 7% (22/291) had an SSI, and 5% (14/291) had a CRBSI. We obtained bacterial growth in 60% (62/109) of LRTI through bronchoalveolar lavage (BAL) of 16% (17/109) and tracheal aspirate of 32% (45/107). Most frequently, isolated bacteria were *Staphylococcus aureus* at 18% (20/109), *Klebsiella pneumoniae* at 10% (11/109), *Enterobacter cloacae* at 8% (9/109), Escherichia *coli* at 7% (8/109), *Pseudomonas aeruginosa* at 6% (7/109), and *Serratia marcescens* at 5% (6/109), among others with minor proportions. Among patients with LRTIs, 55% (61/109) were classified as TAV, 19% (21/109) as hospital-acquired pneumoniae (HAP), and 33% (37/109) as VAP. The median number of days in the hospital before presenting an LRTI was 5 (IQR, 3–9).

Univariate comparison between cohorts with and without LRTIs (Table 2) revealed that patients who had an LRTI were significantly older (median [IQR] 43 years [32–55] vs. 37 years [IQR 25–50], *p* = 0.016) and had more severe head injuries according to GCS (median [IQR], 7 [5–13] vs. 12 [7–14], *p* < 0.01) and AIS head (median [IQR], 4 [3–4] vs. 3 [2–4], *p* < 0.01). APACHE II and SOFA scores were also higher for those patients with LRTIs (median [IQR], 14 [11–18] vs. 12 [6–17], *p* = 0.017) and 5 [4–7] vs. 4 [3–6], *p* < 0.01). In terms of the overall severity of the injury, including other body regions, severity by ISS was worse for the LRTI cohort (median [IQR], 26 [18–33] vs. 22 [14–29], *p* = 0.014). While hospital mortality did not differ between groups (LRTI 18.6% vs. No LRTI 20.1%, *p* = 0.7), ICU LOS was longer in the LRTI group (median [IQR] 12 [9–17] vs. 5 [3–9], *p* < 0.01). Furthermore, hospital LOS was also longer for those with LRTIs when compared to patients without LRTIs (median [IQR] 21 [13–33] vs. 10 [5–18], *p* = 0.01). Time on the ventilator was significantly longer for those with LRTIs (median [IQR] 9 [5–12] vs. 4 [3–7], *p* < 0.01).

**Table 2:** Univariate comparison of baseline characteristics of TBI patients with and without LRTIs.

	**No LRTI (*n =* 182)**	**LRTI (*n* = 109)**	***p*** **-value**
Age	37 (25–50)	43 (32–55)	**0.016**
Sex male (%)	137 (76%)	88 (81%)	0.3
APACHE II median (IQR)	12 (6–17)	14 (11–18)	**0.016**
SOFA median (IQR)	4 (3–6)	5 (4–7)	**0.009**
GCS median (IQR)	12 (7–14)	7 (5–13)	**0.026**
ISS median (IQR)	22 (14–29)	26 (18–33)	**0.015**
AIS head median (IQR)	3 (2–4)	4 (3–4)	**0.016**
MV on admission *n* (%)	140 (76%)	104 (95%)	**<0.001**
Days on MV Median (IQR)	4 (3–7)	9 (5–12)	**<0.001**
Hospital LOS Median (IQR)	10 (5–18)	21 (13–33)	**<0.001**
ICU LOS Median (IQR)	5 (3–9)	12 (9–17)	**<0.001**
Mortality	34 (18.6%)	22 (20.1%)	0.75

MV, mechanical ventilation; LOS, length of stay. Bold values indicate statistically significant.

Potential predictors for the primary outcome (LRTIs) were identified in the bivariate analysis (*p* < 0.1) and included age, ISS, mean blood pressure on admission, GCS, AIS of the head and the thorax, hemoglobin on admission, serum glucose on admission, SOFA, APACHE II, and invasive mechanical ventilation on admission. After multivariable analysis, the following variables remained significantly associated with LRTI (*p* < 0.05): age (OR 1.1, 95% CI, 1.01–1.2), severe TBI (OR 2.7, 95% CI, 1.1–6.9), AIS thorax (OR 1.4, 95% CI, 1.1–1.8), and mechanical ventilation on admission (OR 3.7, 95% CI, 1.1–13.5) (Table 3).

**Table 3:** Multivariate logistic regression analysis for LRTI in TBI patients admitted to ICU.

**Variable**	**OR (95% CI)**	***p value***
Age	1.1 (1.01–1.20)	**0.04**
ISS	1.0 (0.98–1.03)	0.7
MBP on admission	1.0 (0.99–1.03)	0.22
AIS head	1.13 (0.85–1.48)	0.39
AIS thorax	1.42 (1.13–1.79)	**<0.01**
Hemoglobin on admission	1.09 (0.98–1.24)	0.12
Glucose on admission	1.0 (1.0–1.01)	0.06
SOFA admission	0.98 (0.85–1.14)	0.87
APACHE admission	0.93 (0.87–0.99)	0.053
MV on admission	3.7 (1.24–13.5)	**0.026**
GCS ≤8	2.7 (1.11–6.94)	**0.032**

Bold values indicate statistically significant.

In univariate analysis, LRTI was not significantly associated with mortality (OR 1.01, 95% CI 0.88–1.18, *p* = 0.7). Variables associated with mortality (*p* < 0.1) in the univariate analysis included AIS head (OR 1.1, 95% CI 1.07–1.13, *p* < 0.01), ISS (OR 1.01, 95% CI 1.001–1.1, *p* < 0.01), oxygen saturation on admission (OR 0.5, 95% CI, 0.3–0.9, *p* = 0.03), severe TBI (CGS ≤ 8) (OR 1.2, 95% CI 1.08–1.31, *p* ≤ 0.01), SOFA score (OR 1.03, 95% CI 1.01–1.05, *p* < 0.001), APACHE II score (OR 1.02, 95% CI 1.01–1.03, *p* < 0.001), and blood transfusions (OR 1.1, 95% CI 1.01–1.22, *p* = 0.02).

Variables associated with mortality in the multivariable analysis included AIS head, APACHE II score, and transfusion of blood components (Table 4). Multivariate logistic regression also failed to demonstrate a significant association between LRTIs and mortality.

**Table 4:** Multivariate logistic regression analysis for mortality in TBI patients admitted to ICU.

**Variable**	**OR (95% CI)**	***p value***
AIS head	2.25 (1.54–3.47)	**<0.001**
ISS	0.99 (0.95–1.02)	0.56
SatO^2^ on admission	0.4 (0.01–2.1)	0.65
Severe TBI	0.5 (0.18–1.56)	0.25
Transfusions	2.2 (1.10–4.7)	**0.032**
SOFA admission	0.96 (0.82–1.13)	0.87
APACHE admission	2.2 (1.1–4.7)	**<0.001**

Bold values indicate statistically significant.

## Discussion

In this study, we found that a diagnosis of infection was made in almost half of the patients, with the respiratory system being the prevailing source. Among the different types of LRTIs, the most frequent was VAT. Patients who presented an LRTI had a more severe injury to the head and other body regions, had greater disease severity scores, and were older. We identified the following potential predictors for developing LRTIs after TBI in the ICU: age, severe TBI, trauma to the thorax, and being on mechanical ventilation. When comparing outcomes, those who presented with an LRTI stayed longer in the ICU and the hospital and spent more days on mechanical ventilation. However, mortality was not different even after adjusting for age and severity of trauma. This is consistent with a recent large prospective multicenter study that focused on VAP (10).

Previous cohorts had characterized the epidemiology of infections in TBI and found a respiratory source in frequencies as high as 94% of cases (20, 21). We assessed the diagnosis of LRTI through the ATS/IDS guidelines, which include clinical, radiological, and microbiologic criteria. Interestingly in our cohort, the most common type of LRTI was VAT, diagnosed in patients with fever, new or increased sputum production, microbial isolation in tracheal aspirate, and no radiographic evidence of pneumonia (15). Diagnosis and treatment of VAT are controversial and not readily recognized by some, partly due to the difficulty in evaluating infiltrates in a portable X-ray in the ICU. Moreover, severely traumatized patients might have potential confounders like lung contusion and aspiration. However, in the available literature, VAT has been associated with worse clinical outcomes, including progression to VAP, more extended ICU stay, and time on mechanical ventilation (13, 14). Our results underline the importance of a continuous and individualized evaluation of cases at a higher risk of LRTI, always considering differential diagnoses and context when suspecting a respiratory infection. This approach might lead to an earlier and more precise antibiotic prescription for those with a straightforward diagnosis and to avoid the widespread use of antibiotics when not readily indicated. Furthermore, we must strengthen our bundles of care in mechanical ventilation for this population to prevent and decrease LRTIs.

Patients with LRTI in our cohort were older and more severely traumatized. However, after multivariate analysis, the potential risk factors that remained were age, the severity of TBI, thoracic trauma, and being on mechanical ventilation. Other studies have found different variables associated with LRTIs, including blood transfusions, barbiturates infusion, spinal trauma, and ISS (11, 12, 14). Thoracic trauma represents a structural lung disruption that leads to inflammatory changes but does not necessarily end in a respiratory infection (22–24). As differentials, lung contusion, or aspiration warrants a thorough evaluation before diagnosing LRTI and prescribing antibiotics.

We found the severity of TBI as a potential risk factor for LRTIs. Patients with worse TBI might need higher and longer doses of sedation and are more frequently on advanced respiratory support (25, 26). These variables (sedation and mechanical ventilation) have been identified as predictors of LRTI (11). Additionally, we want to highlight that some clinical and preclinical evidence has highlighted that TBI might induce a state of immune depression through inflammatory and autonomic pathways (27, 28). This might facilitate the occurrence of sepsis, not only in the acute phase but also afterward (4). Several animal models have shown evidence that TBI induces cell death mechanisms, including apoptosis, programmed necrosis, or necroptosis (29, 30). Defects in membrane integrity and the release of intracellular components that act like damage-associate molecular patterns (DAMP) induce the further release of cytokines and assembling inflammasomes (8).

The IMPACT study demonstrated that plasma cytokine concentrations are associated with organ dysfunction, mortality, and poor outcomes in TBI [32]. Further research is needed on how this state of immune depression affects outcomes in the TBI population.

Regarding outcomes, patients who suffered from LRTIs had longer ICU and hospital stays and spent longer on respiratory support than those without LRTIs. However, mortality was not significantly different between those cohorts. Previous studies found that LRTI was associated with longer hospital stays, mechanical ventilation, and healthcare costs, coinciding with our results. On the contrary, the results regarding mortality might differ among cohorts (7, 13, 14). One meta-analysis included 15 studies of VAP in TBI and found longer hospitalization but no significant difference in mortality (11). These findings could be related to the awareness of the risk of infection and early antibiotic prescription in cases suspected to have LRTI, which avoids the progression of sepsis and death. However, antibiotics are sometimes not indicated, leading to overuse and increased bacterial resistance. Another explanation for no difference in mortality could be the heterogeneity in the type and severity of TBI, associated injuries, and criteria used for diagnosis of LRTI. These variables might be a difficulty when comparing cohorts and analyzing outcomes.

The main limitation of this study is that patient enrollment was retrospective using electronic medical records. Data collection relied on previous registries, which makes some information challenging to confirm. However, we used standardized definitions and scores to address this limitation and to confirm admission and hospital diagnoses. Outcomes and complications were also evaluated using a thorough medical chart review. Another limitation we acknowledge is that it was a single-center study, which means the generalizability of results can be compromised.

In conclusion, LRTI is a frequent complication in patients with TBI and impacts clinical outcomes. Potential predictors of LRTI were identified; however, these findings are meant to be translated in the future into new hypotheses that deepen our understanding of the immune response in patients with TBI and ultimately lead to the design of novel prognostic and therapeutic tools.

## Data availability statement

The raw data supporting the conclusions of this article will be made available by the authors, without undue reservation.

## Ethics statement

The studies involving human participants were reviewed and approved by Universidad de La Sabana. Written informed consent for participation was not required for this study in accordance with the national legislation and the institutional requirements.

## Author contributions

EC: data analysis, statistical analysis, writing, and edition. MY, EV, and JO: data gathering, review, and edition. LE: data gathering, writing, and edition. NB: data gathering and edition. IM-L: writing, review, and edition. LR: data analysis, writing, review, and edition. All authors contributed to the article and approved the submitted version.
